# Machine learning-enhanced discovery of tsRNA-mRNA regulatory networks: identifying novel diagnostic biomarkers and therapeutic targets in breast cancer

**DOI:** 10.3389/fphar.2025.1640192

**Published:** 2025-07-17

**Authors:** Zhongling Ma, Rui Wang, Ming Yuan, Bo Wang, Li Li, Tianfu Zhao, Xinhan Zhao

**Affiliations:** ^1^ Department of Oncology, The First Affiliated Hospital of Xi’an Jiaotong University, Xi’an, Shaanxi, China; ^2^ Department of Thoracic Surgery, The First Affiliated Hospital of Xi’an Jiaotong University, Xi’an, Shaanxi, China; ^3^ Department of Oncology, Xi’an Chang’an District Hospital, Xi’an, Shaanxi, China

**Keywords:** tsRNA, machine learning, breast cancer, FAM155B, biomarker discovery

## Abstract

**Background:**

Transfer RNA-derived small RNAs (tsRNAs) represent an emerging class of regulatory molecules with potential as cancer biomarkers. However, their diagnostic utility and regulatory mechanisms in breast cancer remain poorly characterized. This study integrates machine learning algorithms with traditional molecular biology approaches to identify tsRNA-based diagnostic signatures and their downstream targets.

**Methods:**

We analyzed miRNA-seq data from 103 matched tumor-normal pairs from TCGA-BRCA as the discovery cohort and GSE117452 as validation. tsRNA profiles were extracted using a custom bioinformatics pipeline. Random forest algorithm was employed to develop a diagnostic model. Correlation analysis and RNAhybrid were used to identify tsRNA-mRNA regulatory relationships. Comprehensive multi-omics analyses including survival, immune infiltration, drug sensitivity, and pathway enrichment were performed for identified targets. Functional validation was conducted in breast cancer cell lines.

**Results:**

We identified 297 differentially expressed tsRNAs and developed a four-tsRNA signature (tRF-21-FSXMSL73E, tRF-20-XSXMSL73, tRF-23-FSXMSL730H, tRF-23-YJE76INB0J) achieving AUC of 0.98 in discovery and 0.82 in validation cohorts. tRF-21-FSXMSL73E showed strong correlation with FAM155B expression. Pan-cancer analysis revealed FAM155B overexpression in multiple malignancies with prognostic significance. FAM155B correlated with immune infiltration, drug resistance, and activation of oncogenic pathways. Functional studies confirmed FAM155B promotes breast cancer proliferation and migration.

**Conclusion:**

Our machine learning approach successfully identified a robust tsRNA diagnostic signature and uncovered the tsRNA-FAM155B regulatory axis as a novel therapeutic target. This integrated methodology provides a framework for accelerating biomarker discovery by combining computational prediction with traditional validation, advancing precision medicine in breast cancer.

## Introduction

The emergence of machine learning algorithms has revolutionized the field of biomarker discovery, enabling researchers to uncover complex molecular patterns that traditional statistical methods might overlook (Duan et al., 2024). In the context of cancer research, this computational revolution has coincided with the discovery of novel classes of regulatory molecules, including transfer RNA-derived small RNAs (tsRNAs), which have emerged as promising candidates for both diagnostic markers and therapeutic targets (Grešová et al., 2022). Transfer RNA-derived small RNAs represent a recently characterized class of small non-coding RNAs generated through specific cleavage of mature or precursor tRNAs (Gu et al., 2022). These fragments, typically ranging from 18 to 40 nucleotides in length, have been implicated in diverse biological processes including gene regulation, stress response, and epigenetic inheritance (Suzuki, 2021). Recent evidence suggests that tsRNAs play crucial roles in cancer development and progression through mechanisms that extend beyond traditional RNA interference pathways (Suzuki, 2021; Xiao et al., 2022). Their stability in biological fluids and disease-specific expression patterns make them particularly attractive as biomarkers for cancer diagnosis and prognosis (Yang et al., 2021).

Breast cancer remains a significant global health burden, representing the most frequently diagnosed cancer among women worldwide (Kawiak, 2024). Despite substantial advances in early detection and treatment strategies, the heterogeneous nature of breast cancer continues to pose challenges for accurate diagnosis and personalized treatment selection (Lüönd et al., 2021; Sunassee et al., 2023). The identification of novel biomarkers that can improve diagnostic accuracy, predict treatment response, and guide therapeutic decisions remains a critical unmet need in breast cancer management (Sabit et al., 2025). The application of machine learning algorithms to high-throughput sequencing data has opened new avenues for biomarker discovery (Fu et al., 2024). Random forest algorithms have demonstrated exceptional performance in handling high-dimensional biological data, capturing non-linear relationships, and identifying combinatorial biomarker signatures (Fu et al., 2024; Ng et al., 2023). These ensemble learning methods are particularly well-suited for tsRNA analysis, where the regulatory effects often involve complex networks of interactions that cannot be adequately captured by univariate statistical approaches (Lu and Wang, 2024).

The integration of machine learning-derived predictions with traditional molecular biology validation represents a powerful strategy for accelerating biomarker discovery and validation (Mottaqi et al., 2025). While computational methods excel at pattern recognition and feature selection in large-scale datasets, experimental validation and mechanistic studies remain essential for establishing biological relevance and clinical utility (Tran et al., 2021). This complementary approach leverages the strengths of both computational and experimental methodologies to provide a more comprehensive understanding of disease mechanisms (Shahwan et al., 2023).

Recent studies have highlighted the potential of tsRNAs as cancer biomarkers, with several reports demonstrating their dysregulation in various malignancies (Jin et al., 2021; Huang et al., 2024). However, the identification of specific tsRNA signatures with robust diagnostic performance and the elucidation of their downstream regulatory targets remain challenging (Wang et al., 2023). The complexity of tsRNA biogenesis, the diversity of their sequences, and their context-dependent functions necessitate sophisticated analytical approaches that can integrate multiple layers of molecular data (Pan et al., 2025; Wang et al., 2025). FAM155B (Family with sequence similarity 155 member B) remains a relatively understudied gene in cancer biology. Previous reports have suggested its potential involvement in endoplasmic reticulum stress response and protein trafficking, but its role in cancer progression has not been comprehensively characterized. Notably, no prior studies have identified regulatory relationships between small non-coding RNAs and FAM155B expression.

In this study, we present a comprehensive approach that combines machine learning-based tsRNA profiling with traditional biomarker discovery and validation methods. By leveraging random forest algorithms to identify diagnostic tsRNA signatures from high-throughput sequencing data, followed by systematic investigation of their regulatory targets and clinical associations, we demonstrate a novel framework for biomarker discovery. Our analysis identified a four-tsRNA signature with exceptional diagnostic performance and revealed FAM155B as a key downstream target with significant implications for breast cancer prognosis and treatment response.

## Methods

### Data sources and patient cohorts

The discovery cohort consisted of breast invasive carcinoma (BRCA) samples from The Cancer Genome Atlas (TCGA) obtained through the Genomic Data Commons (GDC) portal. We specifically selected 103 patients with matched tumor and adjacent normal tissue samples that had available miRNA-seq data. Clinical information including age, gender, pathological stage, overall survival, and progression-free interval was downloaded from the UCSC Xena platform. An independent validation dataset (GSE117452) containing miRNA-seq data from breast cancer patients was obtained from the Gene Expression Omnibus database. This dataset included 50 tumor and 30 normal breast tissue samples processed using similar sequencing protocols. For pan-cancer analysis, we obtained RNA-seq and clinical data for 33 cancer types from TCGA. Normal tissue expression data were acquired from the Genotype-Tissue Expression (GTEx) project, and cancer cell line expression profiles were downloaded from the Cancer Cell Line Encyclopedia (CCLE) database.

## tsRNA extraction and quantification from miRNA-seq data

Raw miRNA-seq FASTQ files were first subjected to quality control using FastQC. Adapter sequences were removed using Cutadapt with minimum length 16 nt, maximum length 50 nt, and minimum quality score 20. A custom bioinformatics pipeline was developed to extract tsRNA sequences from the processed miRNA-seq data. Reference tRNA sequences were downloaded from GtRNAdb for human genome assembly GRCh38, and mature tRNA sequences including 5′leader and 3′trailer sequences were compiled into a custom reference database. Processed reads were mapped to the tRNA reference database using Bowtie2 with parameters specifically optimized for short RNA sequences: -very-sensitive-local mode with--mp 1,1 (mismatch penalty), --rdg 0,1 and--rfg 0,1 (gap penalties), and--score-min G, 1,4 (minimum alignment score function). These parameters were chosen based on preliminary testing with known tsRNA sequences, where default parameters designed for longer reads resulted in significant mapping artifacts and reduced specificity for short tsRNA fragments.

Mapped reads were classified into tsRNA subtypes based on their mapping positions relative to the mature tRNA structure. tRF-5 fragments were defined as those derived from the 5′end of mature tRNA before the anticodon, tRF-3 fragments from the 3′end after the anticodon, tRF-1 fragments from the 3′trailer sequence, and i-tRF as internal fragments spanning the anticodon region. tsRNA expression levels were calculated as reads per million mapped reads (RPM) to normalize for sequencing depth variations. Only tsRNAs with expression ≥1 RPM in at least 10% of samples were retained for analysis, resulting in the identification of 1,113 tsRNA species across all samples.

### Differential expression and statistical analysis

Differential expression analysis between tumor and normal samples was performed using the DESeq2 package in R. Raw count data were normalized using the median of ratios method, and differential expression was assessed using the Wald test. tsRNAs with adjusted p-value <0.05 and absolute log2 fold change >1 were considered significantly differentially expressed. The Benjamini-Hochberg method was used to control the false discovery rate. Volcano plots were generated to visualize the distribution of fold changes and statistical significance using ggplot2. Unsupervised hierarchical clustering was performed on differentially expressed tsRNAs using Euclidean distance and complete linkage method. Heatmaps were generated using the pheatmap package with row-wise z-score normalization.

### Machine learning model development

Random forest classification was implemented using the randomForest package in R to develop a diagnostic model. The model was trained on the TCGA discovery dataset with 10-fold cross-validation to optimize hyperparameters. The number of trees was set to 500, and the number of variables tried at each split (mtry) was optimized through grid search. Feature importance was assessed using mean decrease accuracy, which measures the decrease in model accuracy when a variable is randomly permuted. The top-ranked tsRNAs based on importance scores were selected through recursive feature elimination to identify the minimal set of features maintaining optimal performance. Model performance was evaluated using receiver operating characteristic (ROC) curves, with area under the curve (AUC) as the primary metric. The pROC package was used to calculate AUC values and generate ROC curves. The final diagnostic model consisting of four tsRNAs was validated in the independent GSE117452 dataset. Sensitivity, specificity, positive predictive value, and negative predictive value were calculated at the optimal cutoff determined by maximizing the Youden index.

### tsRNA-mRNA correlation and target prediction

Correlation analysis between tsRNA and mRNA expression profiles was performed using Spearman’s rank correlation coefficient. mRNA expression data from the same TCGA-BRCA samples were obtained and processed using standard RNA-seq analysis pipelines. TPM-normalized expression values were used for correlation analysis. Significant correlations were defined as absolute correlation coefficient >0.3 and adjusted p-value <0.05 after multiple testing correction. RNAhybrid was employed to predict potential tsRNA-mRNA interactions based on thermodynamic stability of RNA duplexes. The minimum free energy threshold was set at −20 kcal/mol to identify high-confidence interactions. This threshold was determined based on a systematic sensitivity analysis evaluating MFE thresholds from −15 to −30 kcal/mol. At −15 kcal/mol, we identified over 3,000 potential targets with relatively weak expression correlations (median |r| = 0.12). At −25 kcal/mol, only 89 targets were identified. The −20 kcal/mol threshold yielded 486 targets with optimal balance of quantity and correlation strength (median |r| = 0.28), which included the FAM155B interaction. Predicted interactions were filtered based on seed region complementarity (positions 2-8 of the tsRNA) and evolutionary conservation of binding sites across species. The combination of expression correlation and computational prediction was used to prioritize potential regulatory relationships.

### Pan-cancer expression analysis

Comprehensive pan-cancer analysis was performed for FAM155B using data from 33 cancer types in TCGA. For each cancer type, differential expression between tumor and normal samples was assessed using the Wilcoxon rank-sum test. To increase the sample size for normal tissues, TCGA tumor data were integrated with GTEx normal tissue data after batch effect correction using the ComBat algorithm. Expression levels were log2-transformed after adding a pseudocount of 1. Cancer cell line expression data from CCLE were analyzed to assess FAM155B expression across different cancer types and tissue origins.

### Clinical association and survival analysis

The relationship between FAM155B expression and pathological stage was examined using the Kruskal-Wallis test followed by Dunn’s *post hoc* test for pairwise comparisons. Overall survival and progression-free interval analyses were conducted using the Kaplan-Meier method. Patients were stratified into high and low expression groups based on the median expression value for each cancer type. The log-rank test was used to compare survival distributions between groups. Cox proportional hazards regression was employed to calculate hazard ratios and 95% confidence intervals, adjusting for available clinical covariates including age, gender, and stage where applicable. Forest plots were generated to visualize hazard ratios across cancer types. To assess the added value of FAM155B in prognostic prediction, we compared nomogram performance with and without FAM155B expression. The C-index improved from 0.672 (clinical variables alone) to 0.708 (clinical variables plus FAM155B), representing a meaningful enhancement in predictive power. This improvement remained consistent across 1,000 bootstrap iterations, confirming the robust added value of FAM155B as a prognostic biomarker.

### Immune infiltration and microenvironment analysis

The CIBERSORT algorithm was applied to estimate the relative abundance of 22 immune cell types in tumor samples based on gene expression profiles. The LM22 signature matrix was used as reference, and only samples with CIBERSORT p-value <0.05 were retained for analysis. Correlations between FAM155B expression and immune cell fractions were calculated using Spearman’s correlation. Tumor microenvironment scores including stromal score, immune score, and ESTIMATE score were calculated using the ESTIMATE algorithm. The relationship between FAM155B expression and various microenvironment features was assessed across cancer types.

### Drug sensitivity analysis

Drug sensitivity data from the NCI-60 cell line panel were obtained from the CellMiner database. The dataset included IC50 values for FDA-approved anticancer drugs and investigational compounds. FAM155B expression levels in NCI-60 cell lines were extracted from the same database. Pearson correlation analysis was performed between FAM155B expression and drug sensitivity values (log10 IC50). Significant associations were defined as p < 0.05 after correction for multiple testing using the Benjamini-Hochberg method.

### Pathway enrichment and co-expression network analysis

Gene Set Variation Analysis (GSVA) was performed using the GSVA package to assess pathway activities in individual samples. The Hallmark gene sets from the Molecular Signatures Database (MSigDB v7.4) were used as reference. Enrichment scores were calculated using the default parameters, and correlations between FAM155B expression and pathway scores were assessed. Gene Set Enrichment Analysis (GSEA) was conducted to identify pathways enriched in FAM155B-high versus FAM155B-low samples, using the median expression as cutoff.

Weighted Gene Co-expression Network Analysis (WGCNA) was performed to identify modules of co-expressed genes. The analysis was conducted on the top 5,000 most variable genes based on median absolute deviation. A soft-thresholding power of 11 was selected based on scale-free topology criteria. The topological overlap matrix was calculated, and hierarchical clustering was performed to identify gene modules. Module eigengenes were correlated with FAM155B expression and clinical traits. Functional enrichment analysis of module genes was performed using clusterProfiler with Gene Ontology and KEGG databases.

### Nomogram construction and validation

A prognostic nomogram was constructed using multivariable Cox regression analysis incorporating FAM155B expression and clinical parameters. Variables were selected based on univariate analysis results and clinical relevance. The final model included age, pathological stage, gender, and FAM155B expression levels. The nomogram was developed using the rms package in R. Model performance was assessed using the concordance index (C-index) and calibration curves. Bootstrap resampling with 1,000 iterations was used for internal validation. Calibration curves comparing predicted and observed survival probabilities were generated for 3-year and 5-year time points.

### Cell culture and transfection

MDA-MB-231 and MDA-MB-453 breast cancer cell lines were obtained from the American Type Culture Collection (ATCC) and cultured in Dulbecco’s Modified Eagle Medium supplemented with 10% fetal bovine serum and 1% penicillin-streptomycin. Cells were maintained at 37°C in a humidified atmosphere containing 5% CO_2_. For FAM155B knockdown, two independent shRNA sequences targeting FAM155B were designed and cloned into the pLKO.1 lentiviral vector. For overexpression studies, the full-length FAM155B coding sequence was cloned into the pcDNA3.1 expression vector. Lentiviral particles were produced in HEK293T cells by co-transfection with packaging plasmids psPAX2 and pMD2.G. Stable cell lines were established by lentiviral transduction followed by puromycin selection.

### Functional assays

Cell proliferation was assessed using the Cell Counting Kit-8 (CCK-8) assay according to the manufacturer’s instructions. Colony formation assays were performed by seeding 800 cells per well in 6-well plates and culturing for 14 days. Colonies were fixed with methanol, stained with crystal violet, and counted. For wound healing assays, cells were grown to confluence and scratched with a pipette tip. Wound closure was monitored at 0-, 6-, and 12-h post-scratch. For xenograft studies, 5 × 10^6^ cells were subcutaneously injected into the flanks of 4–5-week-old female BALB/c nude mice. Tumor volumes were measured every 4 days and calculated using the formula: volume = (length × width^2^)/2. All animal experiments were approved by the Institutional Animal Care and Use Committee.

### Statistical analysis

All statistical analyses were performed using R version 4.0.5. Continuous variables were compared using Student’s t-test for normally distributed data or Wilcoxon rank-sum test for non-parametric data. Multiple group comparisons were performed using one-way ANOVA or Kruskal-Wallis test as appropriate. Correlation analyses were conducted using Spearman’s rank correlation coefficient. All tests were two-sided, and p-values <0.05 were considered statistically significant unless otherwise specified. Multiple testing corrections were applied using the Benjamini-Hochberg method where appropriate. Data visualization was performed using ggplot2 and ComplexHeatmap packages.

## Results

### Machine learning-based identification of diagnostic tsRNA signatures in breast cancer

**FIGURE 1:**
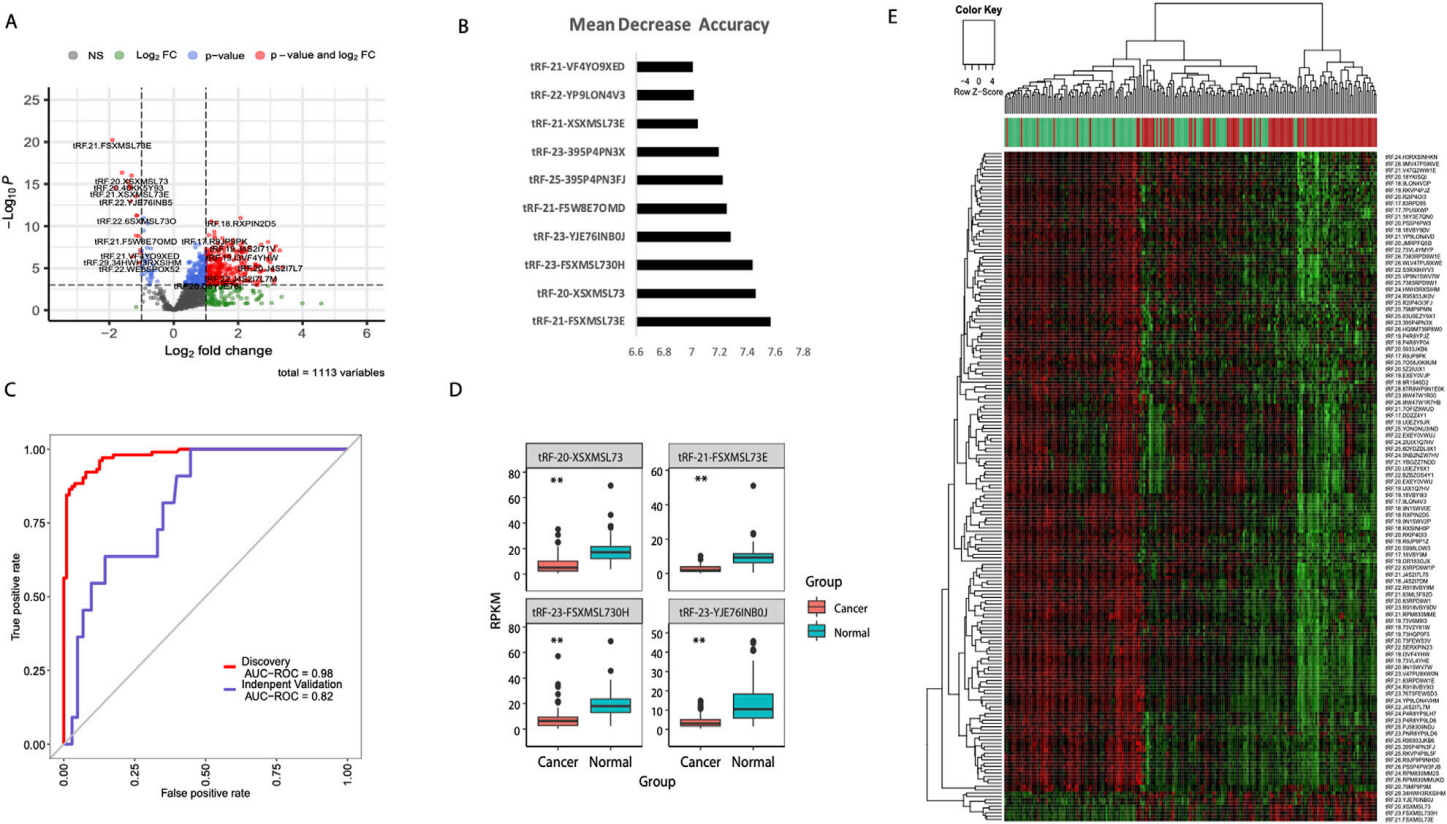
Machine learning-based identification of diagnostic tsRNA signatures in breast cancer. **(A)** Volcano plot showing differential expression of 1,113 tsRNAs between breast tumor (n = 103) and adjacent normal tissues (n = 103) from TCGA-BRCA cohort. Red dots indicate significantly upregulated tsRNAs and blue dots indicate downregulated tsRNAs (adjusted p < 0.05, |log2FC| > 1). **(B)** Random forest feature importance ranking of the top 10 tsRNAs based on mean decrease accuracy. **(C)** Receiver operating characteristic (ROC) curves demonstrating diagnostic performance of the four-tsRNA signature in the TCGA discovery cohort (AUC = 0.98) and independent validation cohort GSE117452 (AUC = 0.82). **(D)** Box plots showing expression levels of the four signature tsRNAs (tRF-21-FSXMSL73E, tRF-20-XSXMSL73, tRF-23-FSXMSL730H, and tRF-23-YJE76INB0J) in cancer versus normal tissues. **P < 0.01; ***P < 0.001. **(E)** Heatmap with hierarchical clustering of differentially expressed tsRNAs clearly separating tumor (red) and normal (green) samples. Color scale represents z-score normalized expression values.

To identify tsRNA-based diagnostic biomarkers for breast cancer, we implemented a comprehensive machine learning workflow using matched tumor and adjacent normal tissue samples from the TCGA-BRCA cohort. From the initial miRNA-seq data, we successfully extracted and quantified 1,113 tsRNA species across 103 paired samples. Differential expression analysis revealed substantial dysregulation of tsRNA profiles in breast cancer tissues, with 297 tsRNAs showing significant differential expression (adjusted p < 0.05, |log2FC| > 1). The volcano plot visualization demonstrated a predominant pattern of tsRNA upregulation in tumor samples, with several tsRNAs exhibiting fold changes exceeding 4-fold (Figure 1A). To develop a clinically applicable diagnostic model, we applied random forest algorithm to the differentially expressed tsRNAs. Through iterative feature selection based on mean decrease accuracy, we identified the top 10 tsRNAs with the highest discriminatory power (Figure 1B). The diagnostic capability of our four-tsRNA signature was evaluated using receiver operating characteristic (ROC) curve analysis. In the TCGA discovery cohort, the model achieved an exceptional area under the curve (AUC) of 0.98, demonstrating near-perfect discrimination between tumor and normal tissues. Importantly, validation in the independent GSE117452 dataset yielded an AUC of 0.82, confirming the robustness and generalizability of our tsRNA signature across different patient populations (Figure 1C). Further optimization revealed that a four-tsRNA signature (tRF-21-FSXMSL73E, tRF-20-XSXMSL73, tRF-23-FSXMSL730H, and tRF-23-YJE76INB0J) achieved optimal diagnostic performance (Figure 1D). Unsupervised hierarchical clustering of differentially expressed tsRNAs clearly separated tumor and normal samples into distinct clusters, indicating robust tsRNA expression signatures associated with malignant transformation (Figure 1E).

### Pan-cancer expression analysis of FAM155B reveals widespread dysregulation

Following the identification of our diagnostic tsRNA signature, we performed correlation analysis between tsRNA and mRNA expression profiles to identify potential regulatory targets. Among the four signature tsRNAs, tRF-21-FSXMSL73E demonstrated the strongest correlation with FAM155B expression (rho = 0.42, p < 0.001). RNAhybrid analysis predicted a thermodynamically stable interaction between tRF-21-FSXMSL73E and FAM155B mRNA (ΔG = −32.5 kcal/mol), prompting comprehensive investigation of FAM155B as a potential therapeutic target.

**FIGURE 2:**
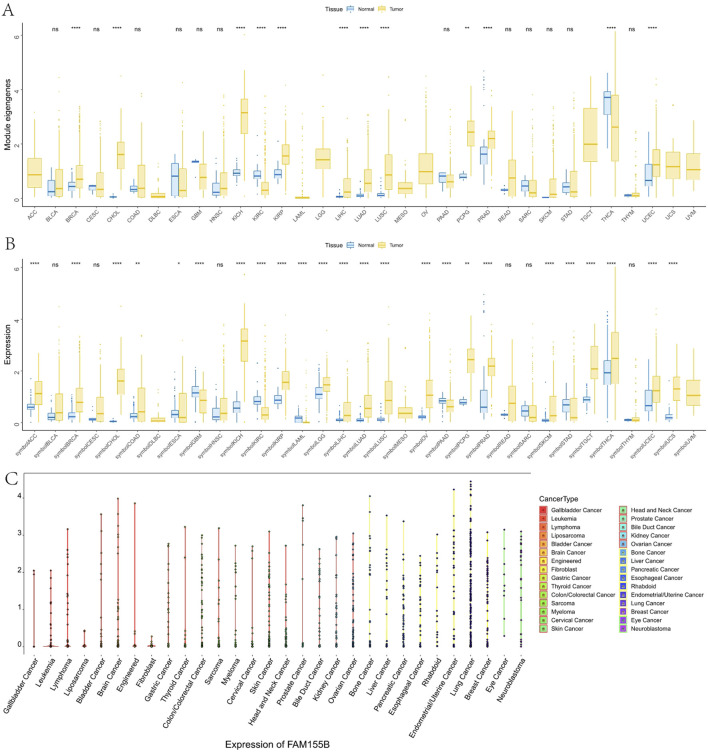
Pan-cancer expression analysis of FAM155B. **(A)** FAM155B mRNA expression levels across 33 cancer types in TCGA dataset. Tumor samples are shown in yellow and normal tissues in blue. **(B)** Comparison of FAM155B expression between tumor and normal tissues integrating TCGA and GTEx databases. **(C)** FAM155B expression levels across 30 different cancer cell line types from the CCLE database, grouped by tissue of origin. *P < 0.05, **P < 0.01, ***P < 0.001.

Pan-cancer analysis using TCGA data revealed significant overexpression of FAM155B across multiple cancer types. FAM155B showed elevated expression in 10 of 33 analyzed cancer types, including breast invasive carcinoma (BRCA), cholangiocarcinoma (CHOL), kidney chromophobe (KICH), kidney renal papillary cell carcinoma (KIRP), liver hepatocellular carcinoma (LIHC), lung adenocarcinoma (LUAD), lung squamous cell carcinoma (LUSC), pheochromocytoma and paraganglioma (PCPG), prostate adenocarcinoma (PRAD), and uterine corpus endometrial carcinoma (UCEC) (Figure 2A). Integration of TCGA and GTEx data confirmed that FAM155B expression was significantly higher in tumor tissues compared to corresponding normal tissues across these cancer types (Figure 2B). Analysis of FAM155B expression in cancer cell lines using CCLE data demonstrated widespread expression across diverse cancer types, with particularly high levels in gallbladder, leukemia, and lymphoma cell lines (Figure 2C). This broad expression pattern suggests that FAM155B may play fundamental roles in cancer cell biology across multiple tissue types.

### FAM155B expression correlates with advanced cancer stage and poor clinical outcomes

**FIGURE 3:**
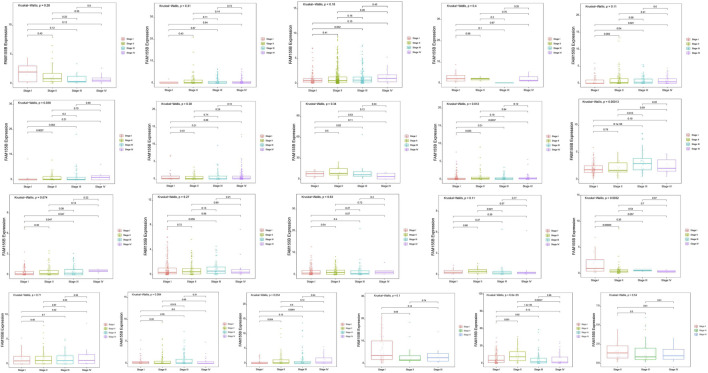
Association of FAM155B expression with pathological stages across multiple cancer types. Box plots showing FAM155B expression levels across different pathological stages in multiple cancer types. From left to right, the first row includes ACC, BLCA, BRCA, CHOL, and COAD; the second row includes ESCA, HNSC, KICH, KIRC, and KIRP; the third row includes LIHC, LUAD, LUSC, MESO, and PAAD; and the fourth row includes READ, SKCM, STAD, TGCT, and UVM. Statistical significance was determined using the Kruskal–Wallis test followed by Dunn’s post hoc test.

**FIGURE 4:**
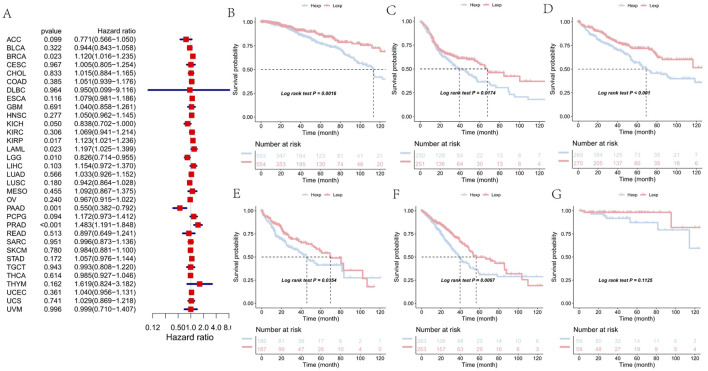
Association of FAM155B expression with overall survival in pan-cancer analysis. **(A)** Forest plot showing hazard ratios for the relationship between FAM155B expression and overall survival across 33 cancer types. **(B–G)** Kaplan-Meier survival curves comparing overall survival between FAM155B high and low expression groups in selected cancer types. P-values were calculated using log-rank test.

To investigate the clinical relevance of FAM155B expression, we examined its association with pathological stage across multiple cancer types. FAM155B expression showed significant positive correlations with tumor stage in several cancers, including esophageal carcinoma (ESCA), kidney renal clear cell carcinoma (KIRC), kidney renal papillary cell carcinoma (KIRP), pancreatic adenocarcinoma (PAAD), stomach adenocarcinoma (STAD), and thyroid carcinoma (THCA) (Figure 3). In breast cancer specifically, FAM155B expression progressively increased from stage I to stage IV, suggesting its involvement in cancer progression and metastasis. Survival analysis revealed that FAM155B expression had significant prognostic implications across multiple cancer types. Forest plot analysis of overall survival (OS) demonstrated that high FAM155B expression was associated with poor prognosis in several cancers, with the most significant associations observed in BRCA (HR = 1.120, p = 0.023), KIRP (HR = 1.123, p = 0.017), LAML (HR = 1.197, p = 0.023), and PRAD (HR = 1.483, p < 0.001) (Figure 4A). Kaplan-Meier analysis confirmed these findings, showing significantly reduced survival in patients with high FAM155B expression (Figures 4B–G).

**FIGURE 5:**
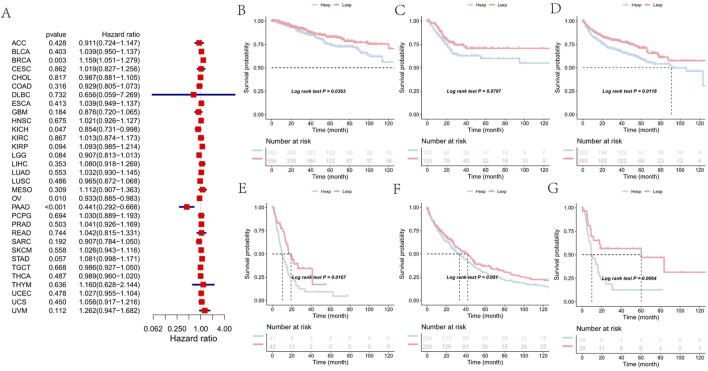
Association of FAM155B expression with progression-free interval in pan-cancer analysis. **(A)** Forest plot showing hazard ratios for the relationship between FAM155B expression and progression-free interval across 33 cancer types. **(B–G)** Kaplan-Meier curves comparing progression-free interval between FAM155B high and low expression groups in selected cancer types. P-values were calculated using log-rank test.

Similar patterns were observed for progression-free interval (PFI), where high FAM155B expression correlated with increased risk of disease progression. The most pronounced effects were seen in BRCA (HR = 1.159, p = 0.003), KIRP (HR = 1.093, p = 0.094), PAAD (HR = 0.441, p < 0.001), and PRAD (HR = 1.041, p = 0.503) (Figure 5A). Kaplan-Meier curves for PFI further validated these associations, demonstrating that FAM155B expression could serve as a prognostic biomarker for disease recurrence (Figures 5B–G).

### FAM155B expression shapes the tumor microenvironment and immune landscape

**FIGURE 6:**
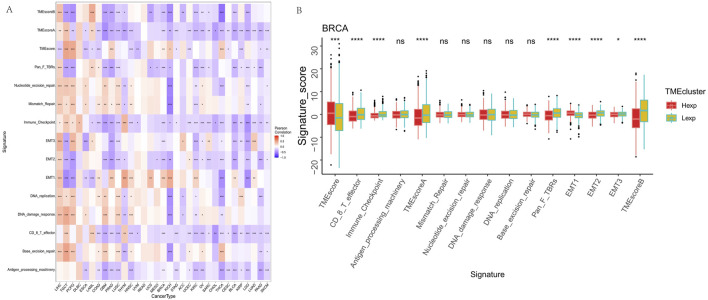
FAM155B expression correlates with tumor microenvironment features. **(A)** Heatmap showing correlations between FAM155B expression and 15 tumor microenvironment processes across multiple cancer types. **(B)** Box plots comparing tumor microenvironment scores between FAM155B high and low expression groups in breast cancer. *P < 0.05; **P < 0.01; ***P < 0.001; ns, not significant.

Given the increasing recognition of the tumor microenvironment’s role in cancer progression and treatment response, we investigated the relationship between FAM155B expression and various microenvironmental features. Correlation analysis across multiple cancer types revealed significant associations between FAM155B expression and key tumor microenvironment processes, including angiogenesis, epithelial-mesenchymal transition, and hypoxia (Figure 6A). In breast cancer specifically, FAM155B expression showed strong positive correlations with TMEscoreB and negative correlations with immune checkpoint-related parameters (Figure 6B).

**FIGURE 7:**
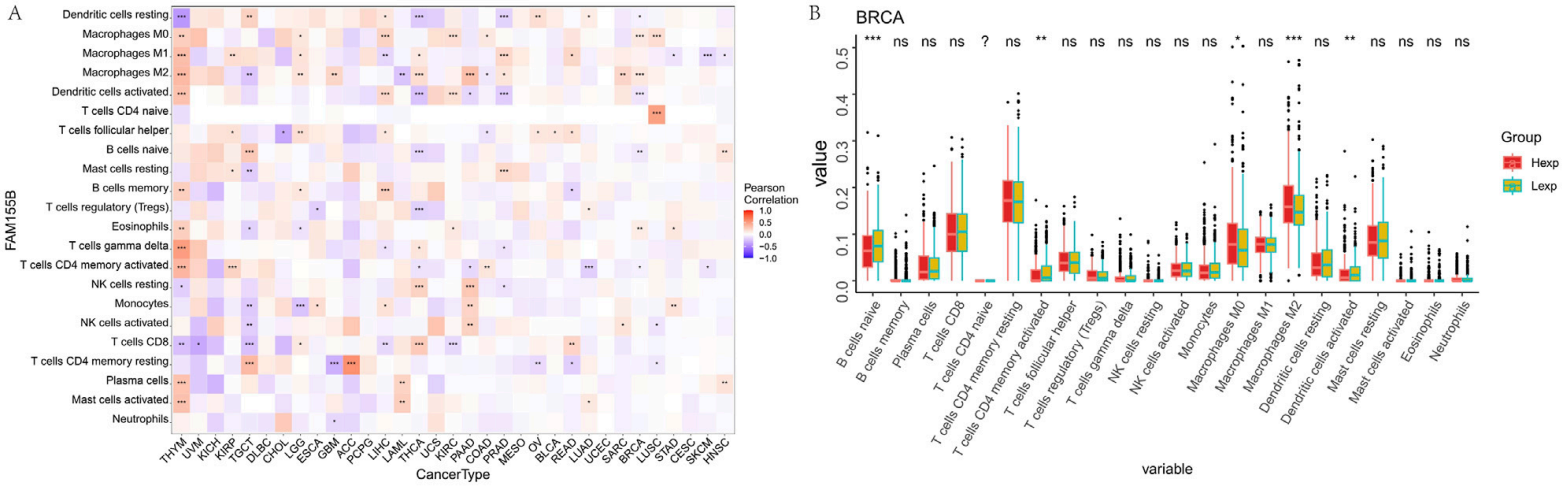
FAM155B expression is associated with immune cell infiltration patterns. **(A)** Heatmap showing correlations between FAM155B expression and infiltration of 22 immune cell types across pan-cancer analysis. **(B)** Box plots showing differences in immune cell proportions between FAM155B high and low expression groups in breast cancer. *P < 0.05; **P < 0.01; ***P < 0.001; ns, not significant.

Detailed immune cell infiltration analysis using CIBERSORT revealed that FAM155B expression was associated with specific immune cell populations across different cancer types. FAM155B showed positive correlations with M0 macrophages in 7 cancer types and M1 macrophages in 10 cancer types, while demonstrating negative correlations with resting dendritic cells in 8 cancer types (Figure 7A). In breast cancer, high FAM155B expression was associated with increased infiltration of M0 macrophages and decreased presence of resting memory CD4^+^ T cells and resting dendritic cells (Figure 7B).

### FAM155B correlates with immune regulatory networks and checkpoint molecules

**FIGURE 8:**
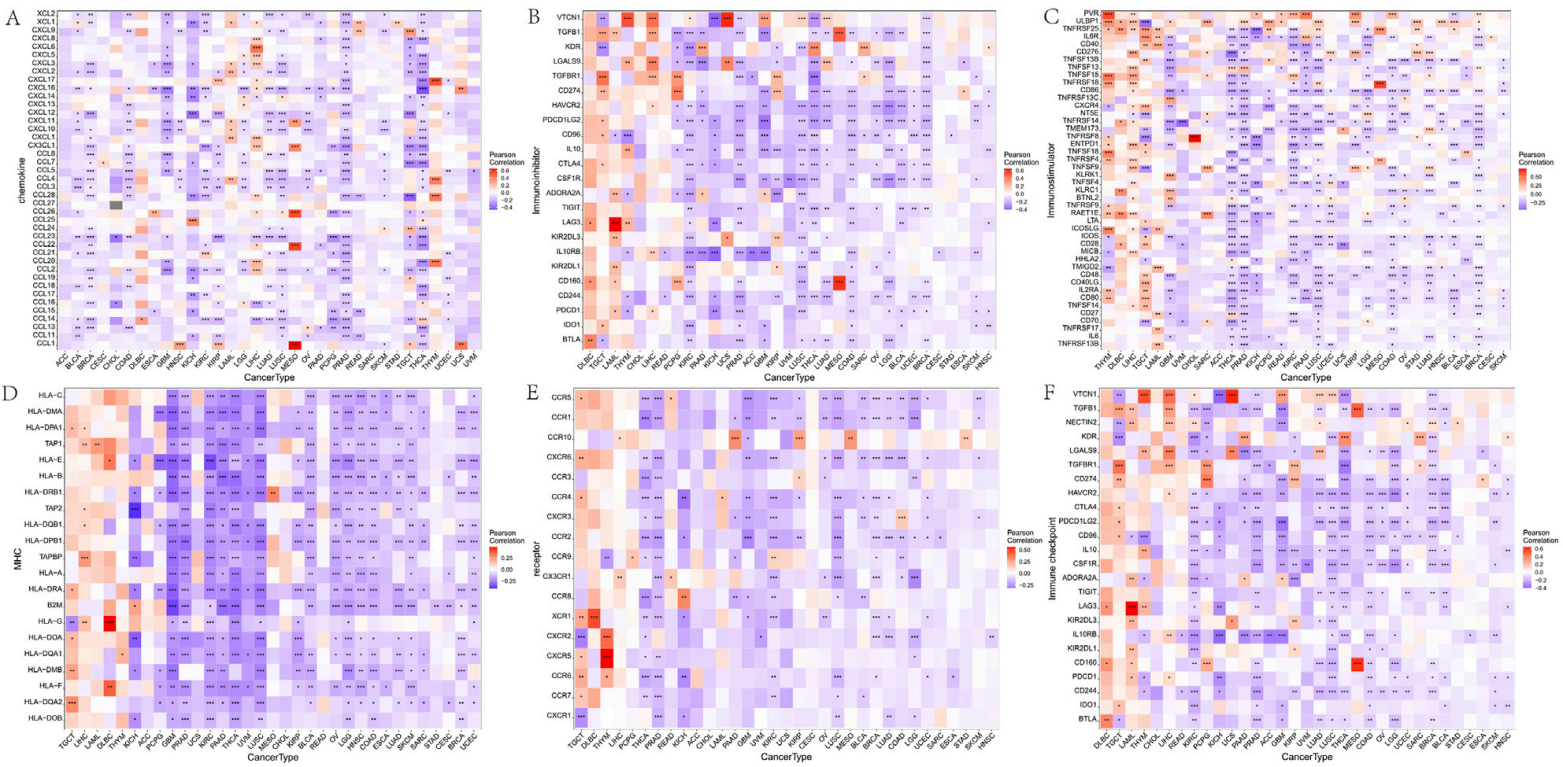
Correlation of FAM155B expression with immune regulatory genes. Heatmaps showing correlations between FAM155B expression and **(A)** chemokine genes, **(B)** immune checkpoint genes, **(C)** immunoinhibitor genes, **(D)** immunostimulator genes, **(E)** MHC genes, and **(F)** receptor genes across multiple cancer types. *P < 0.05; **P < 0.01; ***P < 0.001.

**FIGURE 9:**
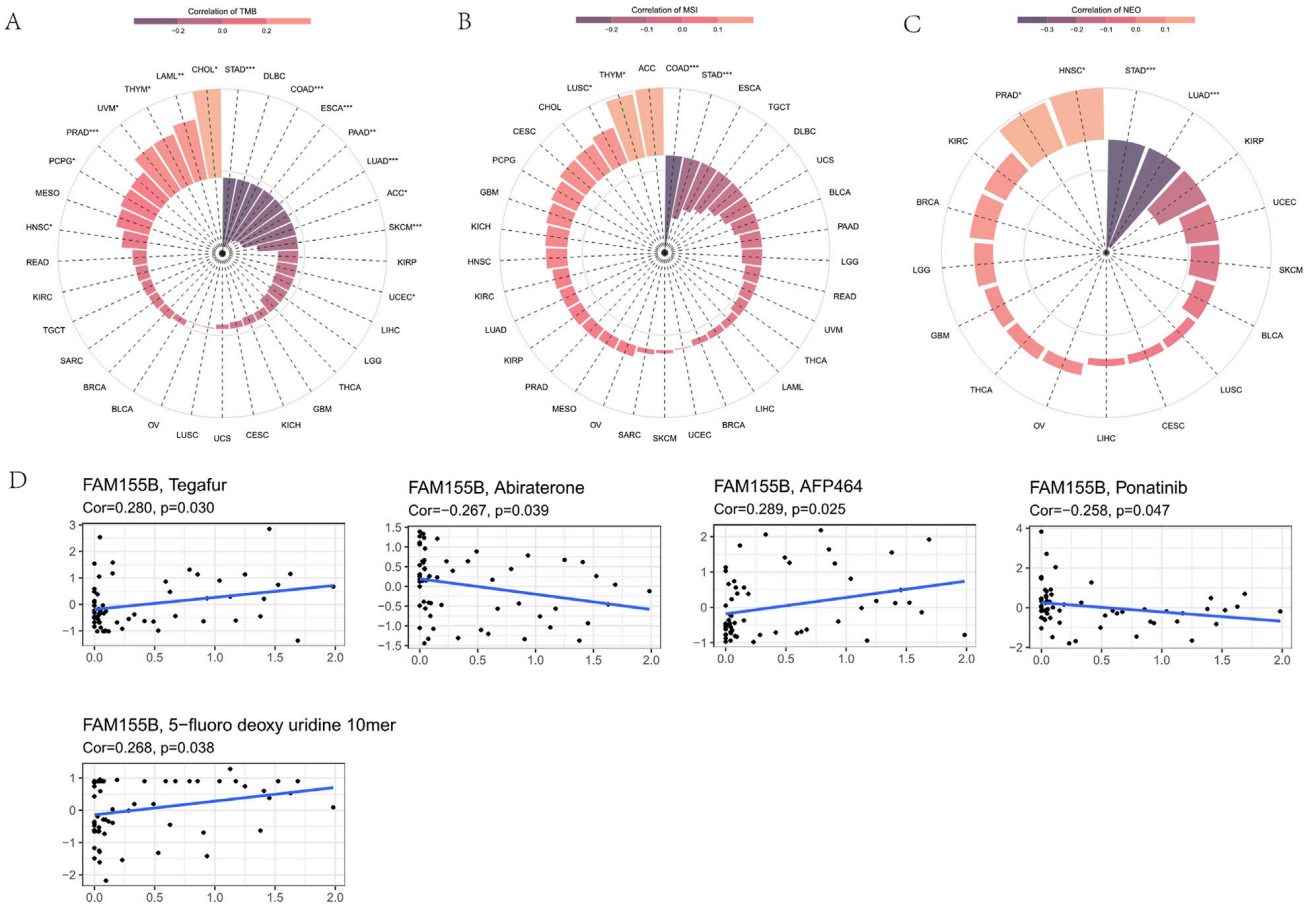
FAM155B expression correlates with TMB, MSI, and drug sensitivity. **(A)** Correlation between FAM155B expression and tumor mutational burden (TMB) across cancer types. **(B)** Correlation between FAM155B expression and microsatellite instability (MSI). **(C)** Correlation between FAM155B expression and neoantigen load (NEO). **(D)** Drug sensitivity analysis showing correlations between FAM155B expression and IC50 values of selected anticancer drugs. *P < 0.05; **P < 0.01; ***P < 0.001.

To further understand FAM155B’s role in immune regulation, we analyzed its correlation with various immune-related gene categories. FAM155B expression showed significant correlations with multiple chemokines, immune checkpoints, immunoinhibitors, immunostimulators, MHC molecules, and their receptors across different cancer types (Figures 8A–F). These widespread correlations suggest that FAM155B may function as a master regulator of tumor immune responses. Analysis of FAM155B expression in relation to tumor mutational burden (TMB), microsatellite instability (MSI), and neoantigen load revealed cancer-specific patterns. FAM155B showed significant positive correlations with TMB in CHOL, LAML, and THYM, while demonstrating negative correlations in PRAD and LUAD (Figure 9A). MSI associations were observed in THYM, LUSC, STAD, and COAD (Figure 9B), and neoantigen correlations were significant in HNSC, PRAD, LUAD, and STAD (Figure 9C).

### FAM155B expression predicts drug sensitivity and resistance patterns

Analysis of drug sensitivity data from the CellMiner database revealed that FAM155B expression was associated with response to multiple anticancer agents. High FAM155B expression correlated with resistance to Tegafur (r = 0.290, p = 0.030), AFP464 (r = 0.289, p = 0.025), and 5-fluoro deoxyuridine 10mer (r = 0.268, p = 0.038), suggesting that FAM155B may contribute to chemotherapy resistance mechanisms. Conversely, FAM155B expression showed negative correlations with Abiraterone (r = −0.267, p = 0.039) and Ponatinib (r = −0.258, p = 0.047), indicating potential sensitivity to these agents (Figure 9D).

### Pathway analysis reveals FAM155B involvement in critical cancer processes

**FIGURE 10:**
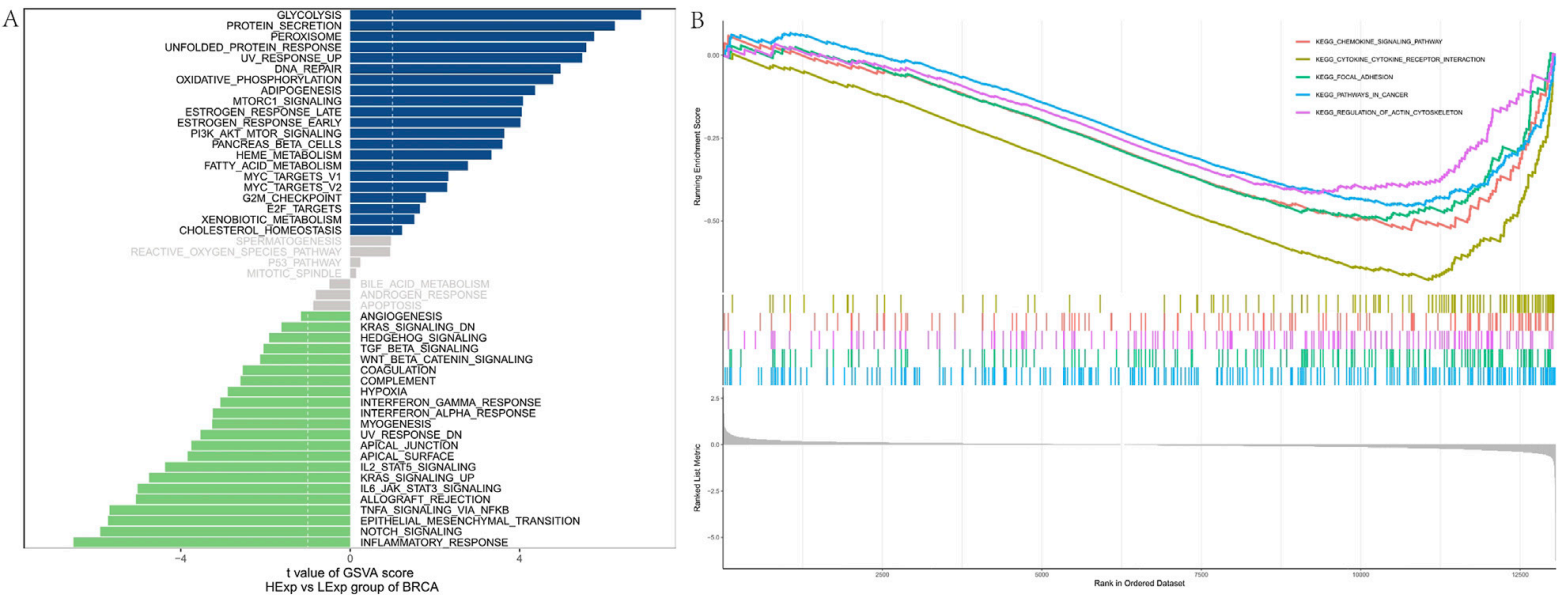
Pathway enrichment analysis of FAM155B in breast cancer. **(A)** GSVA analysis showing correlation between FAM155B expression and hallmark pathway activities. **(B)** GSEA plots showing enrichment of selected pathways in FAM155B high expression group.

To elucidate the biological mechanisms underlying FAM155B’s role in cancer, we performed comprehensive pathway enrichment analysis. GSVA analysis in breast cancer revealed that high FAM155B expression was associated with activation of multiple cancer-related pathways, including glycolysis, protein secretion, peroxisome function, unfolded protein response, UV response, DNA repair, oxidative phosphorylation, adipogenesis, mTORC1 signaling, and estrogen response (Figure 10A). GSEA further confirmed enrichment of these pathways, with particularly strong associations observed for oxidative phosphorylation, DNA repair, and mTORC1 signaling (Figure 10B).

### WGCNA identifies FAM155B-Associated gene modules in breast cancer

**FIGURE 11:**
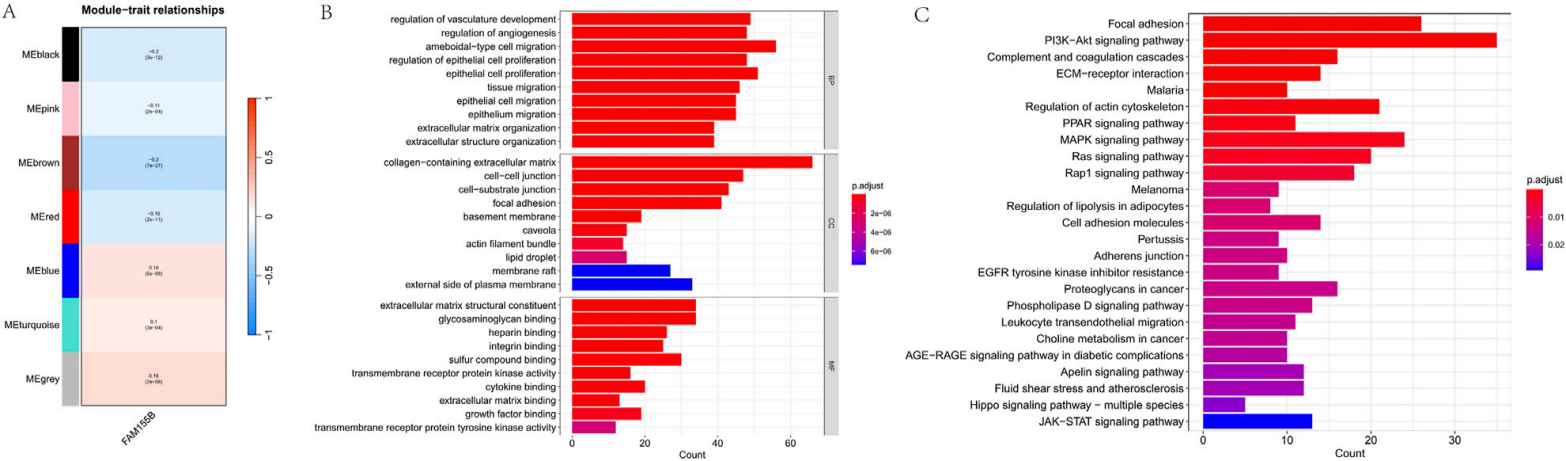
WGCNA identifies FAM155B-associated gene modules in breast cancer. **(A)** Module-trait relationship heatmap showing correlation between gene modules and FAM155B expression. **(B)** GO biological process enrichment analysis of genes in the brown module. **(C)** KEGG pathway enrichment analysis of genes in the brown module.

To identify co-expression networks associated with FAM155B in breast cancer, we performed weighted gene co-expression network analysis (WGCNA). This analysis identified seven distinct gene modules, with the brown module (MEbrown) showing the strongest correlation with FAM155B expression (cor = −0.3, p = 7e-27) (Figure 11A). Functional enrichment analysis of the brown module genes revealed significant enrichment for biological processes related to vasculature development, angiogenesis, and extracellular matrix organization (Figure 11B). KEGG pathway analysis highlighted enrichment for critical signaling pathways including PI3K-Akt, focal adhesion, complement and coagulation cascades, and MAPK signaling (Figure 11C).

### Development of a prognostic nomogram integrating FAM155B expression

**FIGURE 12:**
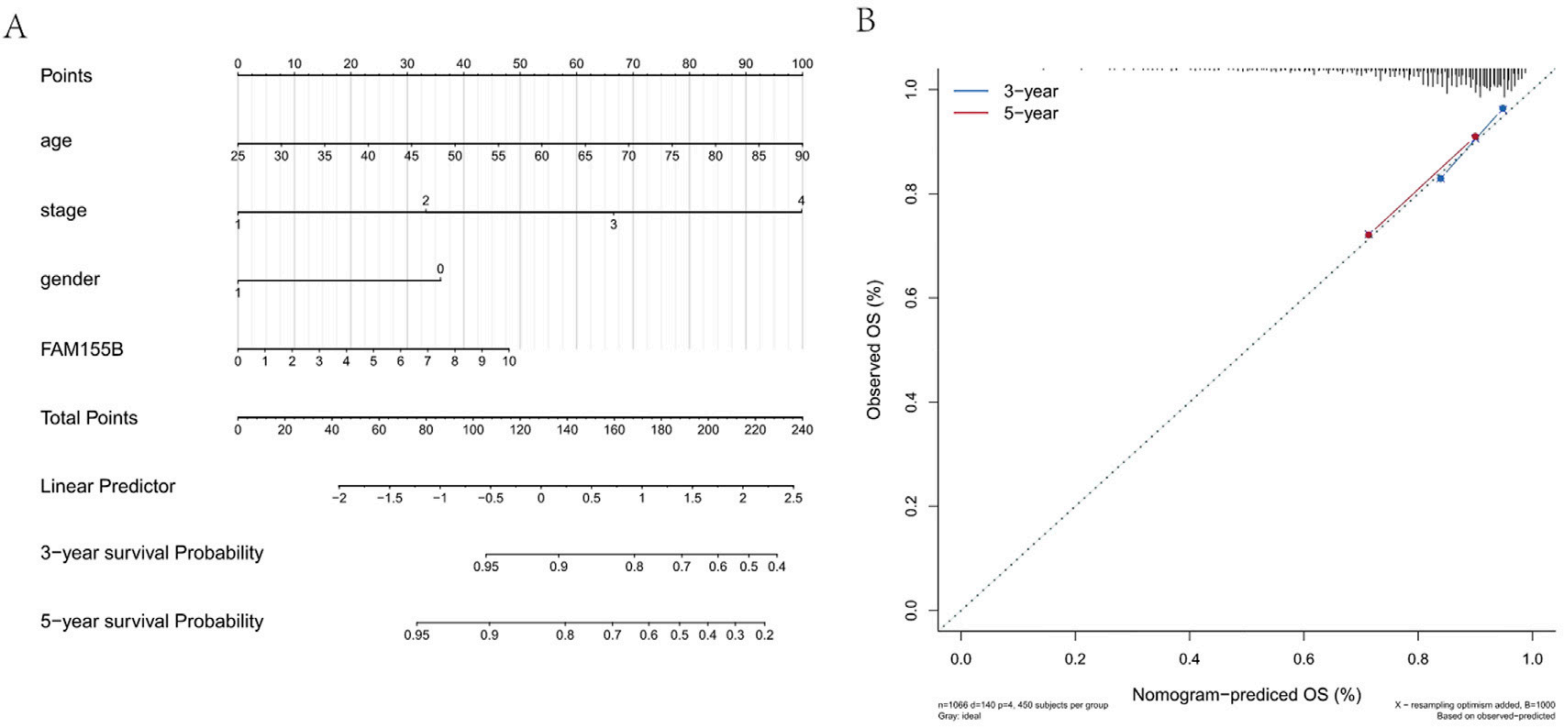
Prognostic nomogram integrating FAM155B expression with clinical parameters. **(A)** Nomogram for predicting 3- and 5-year overall survival in breast cancer patients based on FAM155B expression, age, stage, and gender. **(B)** Calibration curves showing agreement between predicted and observed 3- and 5-year survival probabilities.

To translate our findings into a clinically applicable tool, we developed a prognostic nomogram integrating FAM155B expression with traditional clinical parameters. The nomogram incorporated patient age, tumor stage, gender, and FAM155B expression levels to predict 3-year and 5-year overall survival probabilities (Figure 12A). Calibration curves demonstrated excellent agreement between predicted and observed survival outcomes, with the model showing good discrimination for both 3-year and 5-year predictions (Figure 12B). This integrated model provides a practical framework for incorporating molecular biomarkers into clinical decision-making.

### Functional validation confirms FAM155B’s role in breast cancer progression

**FIGURE 13:**
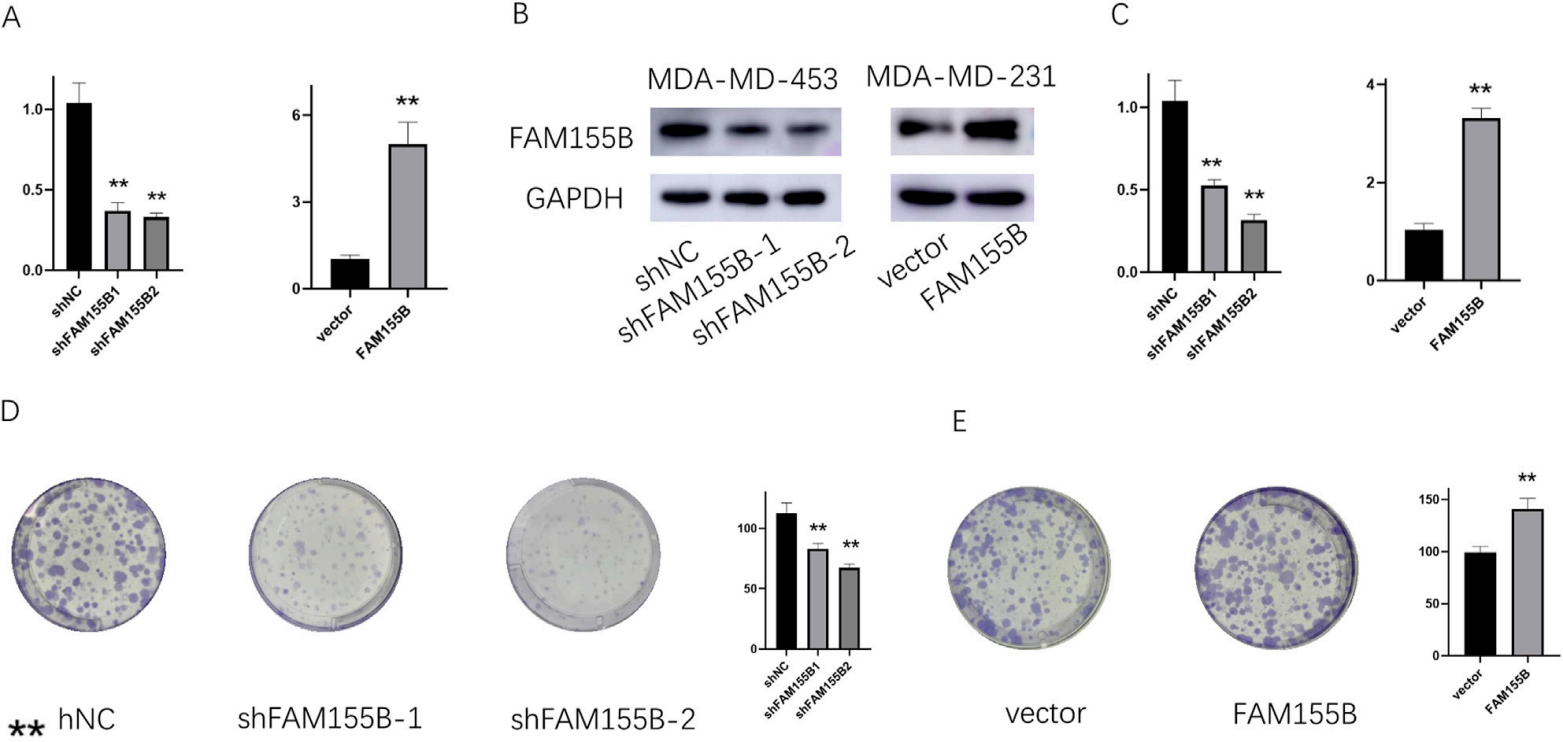
FAM155B knockdown inhibits breast cancer cell proliferation and colony formation. **(A)** qRT-PCR and **(B)** Western blot analysis confirming FAM155B knockdown efficiency in MDA-MB-231 and MDA-MB-453 cells. **(C)** qRT-PCR confirming FAM155B overexpression. **(D)** Colony formation assay showing reduced colony numbers following FAM155B knockdown. **(E)** Colony formation assay showing increased colony numbers following FAM155B overexpression. **P < 0.01.

**FIGURE 14:**
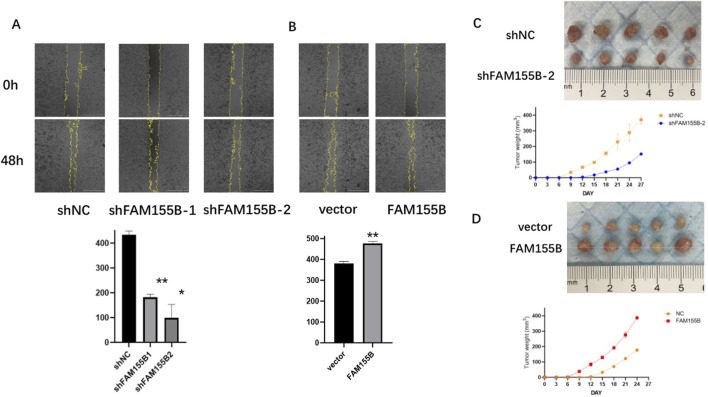
FAM155B promotes breast cancer cell migration and tumor growth. **(A)** Wound healing assay demonstrating reduced migration in FAM155B knockdown cells. **(B)** Wound healing assay showing enhanced migration in FAM155B overexpressing cells. **(C)** Representative images of xenograft tumors from mice injected with control or FAM155B knockdown cells. **(D)** Tumor growth curves and final tumor weights showing reduced tumor growth following FAM155B knockdown. *P < 0.05; **P < 0.01.

To validate the functional significance of FAM155B in breast cancer, we performed loss-of-function and gain-of-function experiments in breast cancer cell lines. Western blot analysis confirmed successful knockdown of FAM155B using two independent shRNA constructs (shFAM155B-1 and shFAM155B-2) and overexpression using a FAM155B expression vector in both MDA-MB-231 and MDA-MB-453 cells (Figures 13A–C). Colony formation assays revealed that FAM155B knockdown significantly reduced the clonogenic potential of breast cancer cells, while overexpression enhanced colony formation capacity (Figures 13D,E). Wound healing assays demonstrated that FAM155B knockdown substantially impaired cell migration, with shFAM155B-1 and shFAM155B-2 reducing wound closure by approximately 60% and 75%, respectively, compared to control cells. Conversely, FAM155B overexpression accelerated wound closure (Figures 14A,B). *In vivo* validation using xenograft models confirmed that FAM155B knockdown significantly reduced tumor growth. Mice bearing tumors from shFAM155B-2 transduced cells showed markedly reduced tumor volumes compared to control groups, with final tumor weights approximately 50% lower than controls (Figures 14C,D). These functional studies validate FAM155B as a critical regulator of breast cancer cell proliferation, migration, and tumor growth, supporting its potential as a therapeutic target.

## Discussion

This study represents a significant advancement in cancer biomarker discovery by successfully integrating machine learning algorithms with traditional molecular biology approaches. Our findings demonstrate that tsRNAs, previously underappreciated regulatory molecules, can serve as highly accurate diagnostic biomarkers for breast cancer when analyzed through sophisticated computational methods. The identification of a four-tsRNA signature with exceptional diagnostic performance (AUC = 0.98) exemplifies how machine learning can extract clinically relevant patterns from complex molecular data that would be challenging to identify through conventional analytical approaches.

The discovery of the tsRNA-FAM155B regulatory axis highlights the power of combining computational predictions with experimental validation. Traditional approaches to biomarker discovery often focus on individual molecules or rely on prior biological knowledge, potentially missing novel regulatory relationships. Our unbiased machine learning approach identified tRF-21-FSXMSL73E as a key diagnostic marker, which subsequently led to the discovery of its association with FAM155B. This finding would have been difficult to achieve through hypothesis-driven research alone, demonstrating the value of data-driven discovery in revealing unexpected biological connections.

The positive correlation between tRF-21-FSXMSL73E and FAM155B expression challenges conventional understanding of small RNA function. While most studies focus on the repressive roles of small RNAs, our findings suggest more complex regulatory mechanisms. This positive correlation might result from several mechanisms: tRF-21-FSXMSL73E could stabilize FAM155B mRNA through competing endogenous RNA networks, regulate upstream transcription factors that control FAM155B expression, or participate in feedback loops that coordinate their expression. These possibilities warrant further mechanistic investigation and highlight the importance of avoiding preconceived notions about molecular interactions when interpreting machine learning predictions. Our WGCNA analysis provides mechanistic insights into this positive correlation. The co-expression of FAM155B with ATF/CREB family transcription factors in the brown module, combined with the sequence complementarity between tRF-21-FSXMSL73E and miRNAs targeting these factors, strongly suggests a competing endogenous RNA mechanism. In this model, tRF-21-FSXMSL73E may function as a molecular sponge that sequesters repressive miRNAs, thereby allowing increased transcription factor activity and subsequent FAM155B upregulation. This mechanism aligns with recent discoveries about tsRNA functions in competing endogenous RNA networks and is supported by our pathway analysis showing enrichment of unfolded protein response pathways.

Our comprehensive validation of FAM155B using traditional biomarker analysis approaches confirms its clinical significance across multiple dimensions. The pan-cancer overexpression pattern suggests that FAM155B may represent a fundamental mechanism in cancer biology rather than a tissue-specific phenomenon. The progressive increase in FAM155B expression with advancing tumor stage, combined with its association with poor survival outcomes, positions it as both a prognostic marker and potential therapeutic target. These findings gained additional credibility through validation across multiple independent datasets and cancer types, demonstrating the robustness of our integrated approach. To our knowledge, this is the first study to comprehensively characterize FAM155B as an oncogenic driver in breast cancer and establish its regulation by tsRNAs. While FAM155B has appeared in various expression profiling studies, its functional role in cancer biology has remained largely unexplored until now.

The relationship between FAM155B and the tumor immune microenvironment reveals another layer of complexity in cancer biology (Zhang and Chen, 2024). Our findings showing positive correlations with immunosuppressive M0 macrophages and negative correlations with antigen-presenting dendritic cells suggest that the tsRNA-FAM155B axis may contribute to immune evasion mechanisms. This observation has important implications for immunotherapy, as FAM155B expression could potentially serve as a biomarker for immune checkpoint inhibitor response or resistance. The integration of machine learning-identified biomarkers with immune profiling represents a promising approach for personalizing immunotherapy strategies.

The drug sensitivity analysis provides actionable insights for precision medicine applications. The association of FAM155B expression with resistance to conventional chemotherapeutic agents like Tegafur and 5-fluorouracil derivatives suggests it may contribute to treatment failure in breast cancer patients (Botticelli et al., 2020). Conversely, the sensitivity to agents like Abiraterone indicates potential therapeutic vulnerabilities that could be exploited. These findings demonstrate how machine learning-guided biomarker discovery can inform treatment selection and identify patients who might benefit from alternative therapeutic strategies.

Our pathway analysis reveals that FAM155B influences multiple hallmark cancer processes, including DNA repair, oxidative phosphorylation, and mTORC1 signaling. This multifaceted involvement suggests that targeting the tsRNA-FAM155B axis could have broad therapeutic effects. The enrichment of DNA repair pathways is particularly intriguing, as it may explain the observed chemotherapy resistance and could indicate synthetic lethal opportunities with DNA-damaging agents or PARP inhibitors. The connection to metabolic pathways like oxidative phosphorylation also suggests potential vulnerabilities to metabolic inhibitors.

The successful validation of our findings through functional experiments strengthens the biological relevance of our computational predictions. The dramatic effects of FAM155B modulation on cancer cell proliferation, migration, and tumor growth confirm its role as a driver of malignant phenotypes. These results provide the necessary biological validation that transforms computational predictions into actionable therapeutic targets. The consistency between *in vitro* and *in vivo* results further supports the potential clinical translation of targeting FAM155B.

From a methodological perspective, our study provides a blueprint for future biomarker discovery efforts. The integration of machine learning with traditional validation approaches leverages the strengths of both methodologies: computational methods excel at pattern recognition and handling high-dimensional data, while experimental approaches provide mechanistic insights and biological validation. This synergistic approach is particularly valuable in the era of big data, where the volume and complexity of molecular information exceed human analytical capacity.

Several limitations should be acknowledged. First, while our correlation and computational analyses suggest regulatory relationships between tRF-21-FSXMSL73E and FAM155B, direct molecular interactions require experimental validation through RNA pulldown or CRISPR-based approaches. Second, the decrease in diagnostic performance between discovery and validation cohorts, while still maintaining good discrimination, highlights the challenges of biomarker generalization across different populations and technical platforms. Third, the mechanisms underlying the positive correlation between tsRNA and target expression require further investigation to fully understand the regulatory networks involved.

Future research directions should focus on several key areas. Mechanistic studies using RNA immunoprecipitation, CLIP-seq, and CRISPR screens could elucidate the precise molecular interactions between tRF-21-FSXMSL73E and FAM155B. Development of tsRNA-based therapeutics, such as antisense oligonucleotides or small molecule inhibitors targeting the tsRNA-FAM155B axis, represents an exciting translational opportunity. Large-scale clinical validation studies are needed to establish the diagnostic utility of our tsRNA signature in clinical settings. Additionally, exploring the role of tsRNAs in other cancer types using our integrated approach could reveal common and tissue-specific regulatory mechanisms.

The clinical implications of our findings are substantial. The four-tsRNA diagnostic signature could be developed into a non-invasive liquid biopsy test for breast cancer detection, given the stability of tsRNAs in biological fluids (Wen et al., 2021). FAM155B expression profiling could guide treatment selection, identifying patients likely to respond to specific therapies. The integration of tsRNA and FAM155B measurements into existing clinical decision-making frameworks, as demonstrated by our nomogram, provides a practical path toward implementation. The translation of our tsRNA signature to liquid biopsy applications faces several important considerations. While our tissue-based analysis provides proof-of-concept, previous studies have demonstrated that tsRNAs are remarkably stable in biological fluids due to their association with protein complexes and extracellular vesicles. The specific tsRNAs in our signature have been detected in circulating biofluid studies, with tRF-21 and tRF-23 fragments particularly enriched in extracellular vesicles. However, critical steps remain before clinical implementation, including: (1) validation of our signature in matched tissue-plasma pairs, (2) optimization of extraction protocols for low-abundance circulating tsRNAs, (3) establishment of reference ranges in healthy populations, and (4) development of specialized protocols to enrich for tsRNA-containing extracellular vesicles. Digital PCR or next-generation sequencing approaches may be required for sensitive detection in liquid biopsies.

In conclusion, our study demonstrates the transformative potential of integrating machine learning with traditional biomarker discovery approaches. The identification of a robust tsRNA diagnostic signature and the discovery of the tsRNA-FAM155B regulatory axis exemplify how computational methods can accelerate biological discovery. As precision medicine continues to evolve, such integrated approaches will be essential for translating the complexity of cancer biology into clinically actionable tools. Our findings not only advance understanding of breast cancer biology but also provide a methodological framework that can be applied across cancer types and molecular data types, ultimately contributing to improved patient outcomes through more precise diagnosis and targeted treatment strategies.

## Data Availability

The data that support the findings of this study are openly available in public repositories. The primary dataset, consisting of miRNA-seq and clinical data for 104 matched tumor and adjacent normal tissue samples from breast invasive carcinoma (BRCA) patients, was obtained from The Cancer Genome Atlas (TCGA), accessible via the Genomic Data Commons (GDC) portal at https://portal.gdc.cancer.gov/. An independent validation dataset was retrieved from the NCBI Gene Expression Omnibus (GEO) under accession number GSE117452 (https://www.ncbi.nlm.nih.gov/geo/query/acc.cgi?acc=GSE117452). Further inquiries can be directed to the corresponding author.
